# Insight into carbapenem resistance and virulence of *Acinetobacter baumannii* from a children’s medical centre in eastern China

**DOI:** 10.1186/s12941-022-00536-0

**Published:** 2022-11-05

**Authors:** Yunfen Zhu, Xin Zhang, Yunzhong Wang, Yunzhen Tao, Xuejun Shao, Yang Li, Wei Li

**Affiliations:** 1 Department of Clinical Laboratory, Children’s Hospital of Soochow University, No. 92, Zhong Nan Street, Industrial Park, Suzhou, China; 2 Institute of Pediatric Research, Children’s Hospital of Soochow University, 215025 Suzhou, Jiangsu China; 3 Clinical Medical College of Pediatrics, Soochow University, 215025 Suzhou, Jiangsu China

**Keywords:** Children, *Acinetobacter baumannii*, Carbapenem resistance, Virulence gene, Epidemiology

## Abstract

Carbapenem-resistant *Acinetobacter baumannii* (CRAB) appeared more frequently in children and caused a great threat to global public health. It is urgent to investigate the carbapenem resistance and virulence of CRAB for clinicians to choose appropriate antibiotics. A retrospective study of 77 nonduplicated CRAB isolates was conducted. The carbapenem resistance and virulence genes were characterized by polymerase chain reaction (PCR) and gel electrophoresis. In the present study, *A. baumannii* mainly came from the intensive care unit and was mostly isolated from sputum samples. The carbapenem resistant rate of *A. baumannii* in 2018–2020 increased significantly compared with that in 2016–2017. All isolates had carbapenem resistant genes. They were highly resistant to a variety of antibiotics but were relatively sensitive to fluoroquinolones and tetracyclines. *bla*VIM and *bla*OXA-23 were detected in all isolates, whereas *bla*OXA-51, *bla*IMP and *bla*NDM were present in 98.70%, 67.53% and 31.17% of isolates, respectively. Notably, 1 isolate *A. baumannii* was identified as multidrug-resistant *A. baumannii* (MDR-AB), and 76 other extensively drug-resistance (XDR) isolates were also detected. Virulence genes were present in 100% of all isolates, including genes in the iron acquisition system (basJ), secretion systems (ompA, plcD), quorum sensing system (abaI) and biofilm formation (csuA). adeH, pgaA, and ptk were present in 98.70%, 98.70% and 94.80% of isolates, respectively. CRAB, which is prevalent in east China, carries a large number of drug resistance and virulence genes. Fluoroquinolones and tetracyclines may be effective antibiotics for the treatment of CRAB infection in children. An in-depth understanding of the resistance and virulence of CRAB is conducive to timely guiding empirical drug use and controlling infection.

**Supplementary Information:**

The online version contains supplementary material available at 10.1186/s12941-022-00536-0.

## Introduction

*Acinetobacter baumannii* (*A. baumannii*) is a common pathogen causing multidrug-resistant nosocomial infections worldwide. Data monitoring studies show that the resistance rate of *A. baumannii* to various antibiotics is rising in many parts of the world [1]. The spread of multidrug-resistant *A. baumannii* (MDR-AB) isolates may be related to the extensive use of broad-spectrum antibiotics, patient cross infection, invasive diagnosis and treatment [2]. With the continuous evolution of MDR-AB isolates, extensively drug-resistant *A. baumannii* (XDR-AB) isolates and even pandrug-resistant *A. baumannii* (PDR-AB) isolates have begun to appear. The problem of nosocomial infection caused by the above pathogen is becoming increasingly serious [3]. Carbapenem antibiotics are generally considered one of the last drugs to treat multidrug-resistant infections. However, its resistance to carbapenem antibiotics is increasing. According to the CDC’s 2019 Antibiotic Resistance Threats Report, carbapenem resistant *A. baumannii* (CRAB) is listed as an urgent threat [4]. The incidence rate of nosocomial infections caused by CRAB is increasing exponentially worldwide. Its complex resistance mechanism and excellent viability limit the choice of treatment after infection, which leads to higher morbidity and mortality^[[5, 6]]^.

The resistance of *A. baumannii* to carbapenem antibiotics is mainly related to the decreased expression of outer membrane channels [7], dysplasia of the discharge pump [8], decreased affinity of penicillin binding protein [9], and production of β-lactamases [10]. The production of β-lactamase is the most common and key mechanism by which CRAB strains exert drug resistance. Based on the Ambler classification, β-lactamase is divided into four molecular classes: A, B, C and D. Class A, C and D enzymes catalyse the hydrolysis of the β-lactamase substrate, forming an intermediate covalent acyl-enzyme complex with a serine residue within the active site. The hydrolysis of class B enzymes is mediated by one or more zinc ions [11–13]. Resistance to CRAB, class B metal-β-lactamases (such as *bla*VIM, *bla*IMP, *bla*NDM, etc.) and class D OXA-carbapenem enzymes (such as *bla*OXA-23, *bla*OXA-24, *bla*OXA-51, etc.) are the most common^[[14, 15]]^, both of which have strong carbapenem hydrolysis ability [16].

In addition to the complex mechanism of drug resistance, CRAB also carries a large number of virulence factors that can easily lead to the deterioration of the disease^[[17, 18]]^. Currently, the virulence determinants of *A. baumannii* are not completely clear, but some studies have shown that its pathogenicity is related to a variety of virulence genes, such as biofilm-forming genes [19], iron acquisition system gene [20], encoding outer membrane protein gene [21], surface glycoconjugates and secretory system-related genes [22].

The complex drug resistance and the role of various virulence factors lead to the serious condition of infected patients and aggravate the death of patients. Therefore, strengthening the monitoring and understanding of CRAB is the key factor in reducing the number of deaths caused by CRAB infection. In recent years, there have been research reports on the virulence and drug resistance of CRAB [23–25], which aim to analyse the outbreak of CRAB in general hospitals. However, due to the differences in action objects, regions and other factors, the characteristics of different strains are also different. There are still few research reports on the correlation between CRAB infection in children in East China, and there is a lack of in-depth analysis of the prevalence, drug resistance and virulence genes of CRAB infection in this region. Therefore, this study aimed to deeply analyse the antimicrobial resistance and virulence genes of CRAB in children of East China Medical Centre and explore the causes of high carbapenem resistance and virulence at the molecular level to provide a basis for clinical anti-infection treatment and nosocomial infection control.

## Materials and methods

### Study site

This study was conducted in Children’s Hospital of Soochow University (CHSU), which is a children’s medical centre in East China and the only provincial tertiary children’s hospital in Jiangsu Province. CHSU has 1500 beds and serves > 70,000 inpatients and > 2 million outpatients annually. This study was approved by the Ethics Committee of CHSU (No. 2021CS158).

### General information

A total of 77 nonduplicated CRAB strains were isolated and taken as the research object from CHSU from January 2016 to December 2020. Judgement criteria of CRAB: resistant to imipenem or meropenem. All nonduplicated *A. baumannii* isolates were confirmed by matrix-assisted laser desorption ionization time-of-flight mass spectrometry (Microflex LT/SH, Germany). The automatic bacterial detection and analysis system (VITEK®2 compact, France) and Kirby-Baue (KB) method were used for the drug sensitivity test. The results were analysed according to the guidelines of the Clinical and Laboratory Standards Institute (CLSI). The quality control strains were *Escherichia coli* (ATCC 25,922), *Pseudomonas aeruginosa* (ATCC 27,853), *Staphylococcus aureus* (ATCC 25,923 and ATCC 29,213), *Enterococcus faecalis* (ATCC 29,212) and *Streptococcus pneumoniae* (49,619), which were purchased from the clinical testing centre of the National Health Commission.

### Detection of virulence and resistance genes

#### Genome extraction

The boiling freezing method for rapid extraction of genomic DNA is currently one of the commonly used methods. The extraction method was described in the literature [26]. The specific steps were as follows: a ring of colonies was picked from the inoculation ring, placed in an EP tube containing 1 mL sterile water, shaken and mixed evenly. Then, the colonies were removed in a 100℃ metal bath for 30 min, placed into a -20℃ refrigerator for 30 min, thawed completely at room temperature, and centrifuged at 15,000 rpm for 5 min. The supernatant was collected and stored. After measuring its concentration and purity by UV spectrophotometry, it was placed in a refrigerator at -20℃ as a template for storage.

#### Genome detection

Focus on the analysis of the carbapenem resistant genome, including class B (*bla*VIM, *bla*IMP, *bla*NDM, *bla*SPM, *bla*BIC, *bla*GIM) and class D (*bla*OXA-23, *bla*OXA-51, *bla*OXA-24, *bla*OXA-2, *bla*OXA-10) [27–31]. Several types of virulence genes were selected for analysis, including biofilm forming genes (adeH, csuA and pgaA), iron acquisition system gene (basJ), encoding outer membrane protein gene (ompA), phospholipase D gene (plcD), capsule positive phenotype gene (ptk) and quorum sensing system regulatory gene (abaI) [32]. Detailed genome sequence information is shown in Table S1. The genome sequence in the experiment was synthesized by Shanghai Branch of Beijing Qingke Biotechnology Co., Ltd. Polymerase chain reaction (PCR) was used to detect the carrier of target genes in the isolates. The PCR products were analysed by 2% agarose gel electrophoresis and positive products were sequenced and aligned with sequences in GenBank using the Basic Local Alignment Search Tool.

### Data analysis

SPSS 20.0 and WHONET v5.6 software (WHO Collaborating Centre for Surveillance of Antimicrobial Resistance, Boston, MA, USA) were used to analyse the data. The counting data were expressed as the number of cases (n) and rate (%). The χ^2^ test was used in univariate analysis. The comparison between groups was carried out by the χ^2^ test, with *p* < 0.05 indicating a statistically significant difference.

## Results

### Epidemiology of CRAB infection

**Fig. 1:**
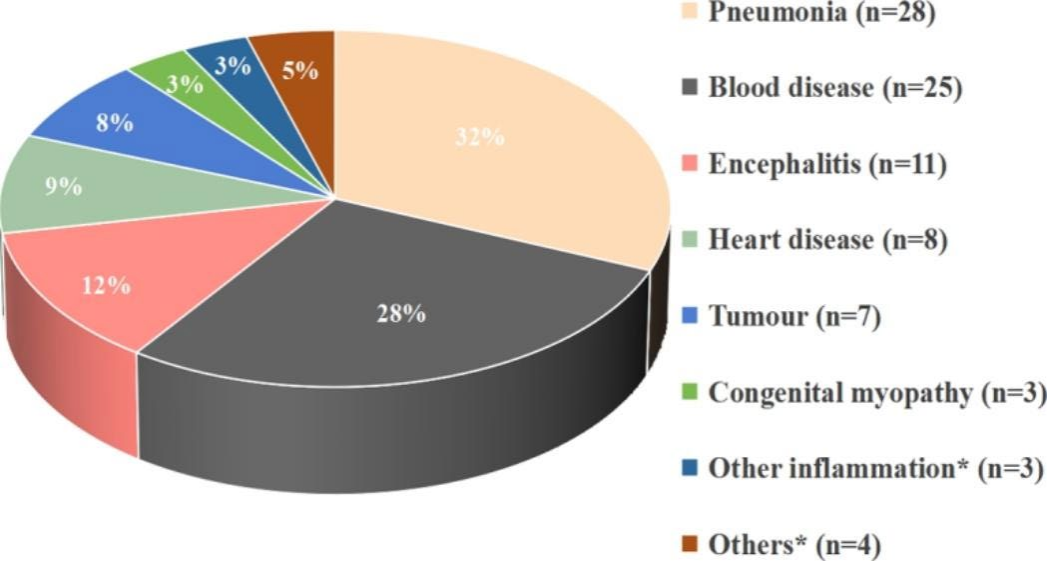
Analysis of basic diseases in 77 children with CRAB infection. *Notes: Other inflammation includes suppurative peritonitis, acute severe pancreatitis, cellulitis; Other basic diseases include dyspnea caused by traffic accidents, foreign body inhalation asphyxia, brain damage, adrenoleukodystrophy. Blood diseases are mainly acute lymphoblastic leukemia

From January 2016 to December 2020, a total of 77 nonduplicated CRAB strains were isolated from patients, including 48 isolates from males (62.34%) and 29 isolates from females (37.66%). Among 77 isolates of CRAB, 65 strains were isolated from the intensive care unit (ICU), 60 isolates were derived from sputum, and 71 strains were isolated from surviving patients. See Table 1 for other information. Among 77 children with CRAB infection, the basic diseases of pneumonia and blood diseases were the most common. The details are shown in Fig. 1.

**Table 1:** Epidemiology of CRAB in this study [n (%)]

Classification	ICU (n = 65)	Other wards^*^ (n = 12)	χ^2^	*p*	OR	95% CI
**Specimen, n (%)**						
Sputum (n = 60)	55 (91.67)	5 (8.33)	10.861	**0.001**	7.700	2.035–29.138
Blood (n = 6)	3 (50.00)	3 (50.00)	5.858	**0.016**	0.145	0.025–0.832
Alveolar lavage fluid (n = 5)	4 (80.00)	1 (20.00)	0.079	0.778	0.721	0.074–7.076
Urine (n = 3)	2 (66.67)	1 (33.33)	0.747	0.387	0.349	0.029–4.188
Others specimen (n = 3)	1 (33.33)	2 (66.67)	6.192	**0.013**	0.078	0.006–0.943
**Age, n (%)**						
0 ~ < 3 Y (n = 34)	30 (88.24)	4 (11.76)	0.675	0.411	1.714	0.469–6.262
3 ~ < 14 Y (n = 39)	31 (79.49)	8 (20.51)	1.459	0.227	0.456	0.125–1.665
14 ~ < 18 Y (n = 4)	4 (100.00)	0 (0)	0.779	0.377	0.938	0.882–0.999
**Survival state, n (%)**						
Survival (n = 71)	60 (84.51)	11 (15.49)	0.006	0.939	1.091	0.116–10.260
Death (n = 6)	5 (83.33)	1 (16.67)	0.021	0.884	0.846	0.090–7.949
**Gender, n (%)**						
Male (n = 48)	40 (83.33)	8 (16.67)	0.113	0.736	0.800	0.218–2.936
Female (n = 29)	25 (86.21)	4 (13.79)	0.113	0.736	1.250	0.341–4.587
**Length of stay, n (%)**						
0 ~ < 15 d (n = 5)	5 (100.00)	0 (0)	0.987	0.320	0.923	0.861–0.990
15 ~ 30 d (n = 36)	30 (83.33)	6 (16.67)	0.060	0.806	0.857	0.250–2.939
> 30 d (n = 36)	30 (83.33)	6 (16.67)	0.060	0.806	0.857	0.250–2.939

^*Notes: Other wards include the departments of hematology, neonatology, urology, respiratory, general sugery; Other specimens include tissue and pharynx. Abbreviations: d, days; Y, years; OR, odds ratio; 95% CI, 95% confidence interval^

### Analysis of the carbapenem resistance rate of *A. baumannii* from 2016 to 2020

**Fig. 2:**
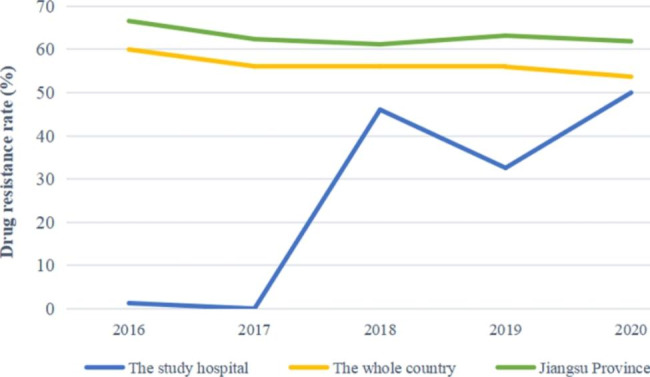
The drug resistance rate of *A. baumannii* in the study hospital, China, and Jiangsu Province between 2016 to 2020. *Notes: The drug resistance rate of *A. baumannii* in the whole country comes from National Drug Resistance Monitoring Network. The member units that report the data are mainly tertiary hospitals, which report the monitoring data from October of the current year to September of the next year every year. The principle of retaining the first strain of the same bacteria in the same patient is to eliminate duplicate strains

According to the data of the National Drug Resistance Monitoring Network (http://www.carss.cn/), during the five years from 2016 to 2020, the carbapenem resistance rate of *A. baumannii* in China fluctuated between 56% and 60%, slightly lower than that in Jiangsu Province. However, the drug resistance rate in 2018–2020 increased significantly compared with that in 2016–2017 in the local children’s medical centre (Fig. 2).

### Antimicrobial susceptibility testing

Seventy-seven isolates of CRAB exhibited different patterns of resistance to various antibiotics, but in general, the drug resistance rate was generally high, as showen in Table 2. They showed a 100% resistance rate to carbapenems (imipenem), extended-spectrum cephalosporins (cefotetan, ceftazidime, ceftriaxone, cefepime), enzyme inhibitor complexes (ampicillin/sulbactam, piperacillin/tazobactam, ticarcillin/clavulanic acid, cefoperazone/sulbactam) and penicillins (ampicillin). They showed low resistance to fluoroquinolones (levofloxacin) and tetracyclines (minocycline). The resistance rate to tigecycline was only 2.6%.

**Table 2:** The antimicrobial resistance of CRAB in this study [n (%)]

Antibiotics	Number of strains detected		
	**Resistance**	**Intermediary**	**Sensitivity**
**Carbapenems**			
Imipenem	77 (100)	0 (0)	0 (0)
**Extended spectrum cephalosporin**			
Cefotetan	77 (100)	0 (0)	0 (0)
Ceftazidime	77 (100)	0 (0)	0 (0)
Ceftriaxone	77 (100)	0 (0)	0 (0)
Cefepime	77 (100)	0 (0)	0 (0)
**Enzyme inhibitor complex**			
Ampicillin/sulbactam	77 (100)	0 (0)	0 (0)
Piperacillin/tazobactam	77 (100)	0 (0)	0 (0)
Ticarcillin/clavulanic acid	77 (100)	0 (0)	0 (0)
Cefoperazone/sulbactam	77 (100)	0 (0)	0 (0)
**Penicillins**			
Ampicillin	77 (100)	0 (0)	0 (0)
**Aminoglycosides**			
Gentamicin	73 (94.81)	4 (5.19)	0 (0)
Amikacin	72 (93.51)	0 (0)	5 (6.49)
Tobramycin	70 (90.91)	1 (1.30)	6 (7.79)
**Sulfonamides**			
Compound sulfamethoxazole	72 (93.51)	0 (0)	5 (6.49)
**Fluoroquinolones**			
Ciprofloxacin	76 (98.70)	0 (0)	1 (1.30)
Levofloxacin	17 (22.08)	59 (76.62)	1 (1.30)
**Tetracyclines**			
Minocycline	18 (23.38)	54 (70.13)	5 (6.49)
Tigecycline	2 (2.60)	68 (88.31)	7 (9.09)

### Detection of the carbapenem resistance genes

Resistance to CRAB, class B metal-β-lactamases and class D OXA-carbapenem enzymes are the most common. As seen in Table 3, *bla*VIM and *bla*OXA-23 were detected in all isolates, and *bla*OXA-51 was present in 98.70% of isolates. The detection rates of *bla*IMP and *bla*NDM were 67.53% and 31.17%, respectively. See Table S2 for the details of carbapenem resistance genes carried by 77 CRAB isolates.

**Table 3:** Analysis of resistance gene detection in this study

Resistance genes	Number of strains detected			Detection rate (%)
	**ICU**	**Other wards** ^*^	**Total**	
**Class B**				
*bla* VIM	65	12	77	100
*bla*IMP	47	5	52	67.53
*bla*NDM	20	4	24	31.17
*bla*AIM	0	0	0	0
*bla*SPM	0	0	0	0
*bla*BIC	0	0	0	0
*bla*GIM	0	0	0	0
**Class D**				
*bla*OXA-23	65	12	77	100
*bla*OXA-51	64	12	76	98.70
*bla*OXA-2	0	0	0	0
*bla*OXA-10	0	0	0	0
*bla*OXA-24	0	0	0	0

^*Notes: Other wards include the Departments of Haematology, Neonatology, Urology, Respiratory, and General sugery^

### Detection virulence genes

The carrying rates of all virulence genes were more than 90%. The genes basJ, ompA, plcD, abaI and csuA were present in 100% of all isolates. The adeH, pgaA, and ptk genes were present in 98.70%, 98.70% and 94.80% of all isolates, respectively (Table 4). The study also found that 73 isolates carried 8 virulence genes, 3 isolates carried 7 virulence genes and 1 isolate carried 5 virulence genes (Table S3).

**Table 4:** Analysis of virulence gene detection in this study

Virulence genes	Number of strains detected (n)				Detection rate (%)
	**ICU**	**Other wards** ^*^		**Total**	
Outer membrane protein gene					
ompA	65		12	77	100
Iron acquisition system gene					
basJ	65		12	77	100
Phospholipase D gene					
plcD	65		12	77	100
Quorum sensing system regulating genes					
abaI	65		12	77	100
Biofilm forming gene					
csuA	65		12	77	100
adeH	64		12	76	98.70
pgaA	64		12	76	98.70
Capsule positive phenotype gene					
ptk	61		12	73	94.80

^*Notes: Other wards include the Departments of Haematology, Neonatology, Urology, Respiratory, and General sugery^

## Discussion

Carbapenem antibiotics are the “Ace Killer” of gram-negative bacteria because of their strong selectivity and low toxicity to host cells. However, with the wide use of antibiotics, XDR-AB shows an increasing trend, and the effect of carbapenem antibiotics is also gradually reduced. CRAB carries a large number of virulence genes, has strong invasiveness and often causes severe pneumonia, meningitis, and endocarditis [33]. This seriously threatens the lives of people with low immune function and suffering from serious underlying diseases and has become a public health threat all over the world. The results of this study showed that among 77 isolates of CRAB, 65 isolates were mainly from ICU, accounting for 84.41%, which is consistent with reports that CRAB is mainly isolated from ICU [5, 23, 34]. There were differences in CRAB detection in sputum and blood samples between ICU departments and non-ICU departments (*p* < 0.05). The widespread prevalence of CRAB in the ICU may be related to the large number of severe patients with low immunity, catheter and mechanical ventilation operations, and the wide use of antibiotics [10]. Therefore, for ICU patients, medical workers need to strengthen aseptic awareness, standardize hand hygiene, block transmission routes and avoid cross-infection. The isolates were mainly from sputum samples, accounting for 77.92% (60/77). This may be due to the patient’s invasive diagnosis and treatment and the fact that *A. baumannii* itself is a respiratory colonization bacterium. The proportion of CRAB in males was slightly higher than that in females. From the perspective of age group, the proportion of children aged 14 ~ < 18 years has a relatively low risk of infection, accounting for only 5.19%. This may be related to the improvement of children’s immunity at this stage. The hospitalization time was generally long, and only 5 cases were less than 15 days. Survival state analysis found that 6 death cases were mainly patients with serious basic diseases (such as leukaemia and tumour). Among them, 4 strains of CRAB were isolated from sputum and 2 from blood. In the sputum sample group, mortality accounted for 6.67% (4/60), while in the blood sample group, mortality accounted for 33.33% (2/6), which is consistent with the mortality of 28%~43% reported by Chopra et al. [35]. It can be seen that the mortality of blood infection caused by CRAB is high. Special attention should be given to CRAB detected by the blood circulation system. Clinical response measures should be taken in time to do a good job in the isolation and protection of the same department. As shown in Fig. 1, the basic diseases of 77 cases were mainly pneumonia, blood disease, encephalitis, heart disease and tumours. In the clinic, special attention should be given to the infection of children with the above basic diseases to achieve early prevention, early detection and early treatment.

National Drug Resistance Monitoring Network data show that the carbapenem resistance rate of *A. baumannii* fluctuated between 61.20 and 66.60% in Jiangsu Province from 2016 to 2020, which was higher than the national average rate of carbapenem resistance (56-60%). Some studies on the carbapenem resistance of gram-negative pathogens in children showed that the detection rate of CRAB was as high as 41.6-76.8%^[[36, 37]]^. As shown in Fig. 2, the results of the local children’s medical centre show that the carbapenem resistance rate in 2018–2020 (32.58-50%) increased significantly compared with that in 2016–2017 (0-1.27%). The high detection rate of CRAB suggested that there may be a pandemic of this kind of pathogen in the hospital, which needs to be given great attention by clinical medical staff. The antimicrobial susceptibility testing results showed that 77 isolates of CRAB exhibited different patterns of resistance to various antibiotics. Seventy-seven isolates of CRAB showed a 100% resistance rate to carbapenems (imipenem), extended-spectrum cephalosporins (cefotetan, ceftazidime, ceftriaxone, cefepime), enzyme inhibitor complexes (ampicillin/sulbactam, piperacillin/tazobactam, ticarcillin/clavulanic acid, cefoperazone/sulbactam), and penicillins (ampicillin) and more than 90% resistance rates to gentamicin, amikacin, tobramycin, compound sulfamethoxazole, ciprofloxacin. However, the drug resistance rates of isolates to levofloxacin, minocycline and tigecycline were low, accounting for 22.08%, 23.38% and 2.6%, respectively. Among them, 77 isolates showed lower resistance rates to tigecycline, and the intermediary and sensitivity rates of tigecycline were 88.31% and 9.09%, respectively. The low resistance rate of tigecycline is consistent with a report of *A. baumannii* infection in children [37]. However, such drugs may be related to the kidney and ototoxicity to children. With the increasingly limited choice of antibiotics, tigecycline is gradually being used in the treatment of *A. baumannii* infection in children [38]. The combination of tigecycline with carbapenems or other antibiotics is one of the best choices for the treatment of patients with MDR-AB infection at present. Notably, 1 MDR-AB isolate and 76 XDR-AB isolates were also detected.

The wide distribution of CRAB is due to multiple mechanisms with multiple genetic determinants. The production of class B metal-β-lactamase and class D oxacillinase enzymes are the most common mechanisms of drug resistance to CRAB, among which class D oxacillinases are the most widely used. It has been reported that *bla*OXA-23 and *bla*OXA-51 are the most common genes causing CRAB in class D [2, 39], which is basically consistent with the results of this study. *bla*OXA-23 and *bla*OXA-51 were present in 100% and 98.70% of isolates, respectively. The *bla*OXA-23 carbapenemase produced is mostly plasmid mediated, which is very easy to spread laterally among different strains, resulting in the spread of carbapenem resistance. It has been reported that overexpression of *bla*OXA-51 can lead to carbapenem antibiotic resistance [40]. It belongs to the class B metal-β-lactamases blaVIM, *bla*IMP and *bla*NDM, which were present in 100%, 67.53% and 31.17% of isolates, respectively. As reported, 25% of CRAB isolates in Asia have been found to contain *bla*NDM [41], which is close to the results of this study. A study showed that the class B metal-β-lactamases genes detected in *A. baumannii* are mainly *bla*VIM and *bla*NDM [42]. In disagreement with this finding, Kongthai et al. found that the detection rate of *bla*NDM was 3.7%, and *bla*VIM and *bla*IMP were not detected [24]. The above results may indicate that the horizontal transmission of *bla*OXA-23, *bla*VIM, and *bla*OXA-51 genes is the main reason for the transmission of carbapenem resistance of *A. baumannii* in this area. Further analysis of the types of carbapenem resistant genes carried by 77 isolates showed that 16 isolates (accounting for 20.78%) were detected with 5 genes of *bla*OXA−23/*bla*OXA-51/*bla*VIM/*bla*IMP/*bla*NDM, 35 isolates (45.45%) with 4 genes of *bla*OXA−23/*bla*OXA-51/*bla*VIM/*bla*IMP, 7 isolates (9.09%) with 4 genes of *bla*OXA−23/*bla*OXA-51/*bla*VIM/*bla*NDM, 1 isolate (1.30%) with 4 genes of *bla*OXA−23/*bla*VIM/*bla*IMP/*bla*NDM and 18 isolates (23.38%) with 3 genes of *bla*OXA−23/*bla*OXA-51/*bla*VIM. The above research showed that class B metal-β-lactamases and class D oxacillinase enzymes coexist in the resistance mechanism of CRAB in local children’s medical centre.

In recent years, research on *A. baumannii* has mainly focused on the mechanism of drug resistance and epidemic characteristics, and there is less research on the mechanism of virulence. Currently, virulence factors found in *A. baumannii* mainly include the biofilm forming system, iron acquisition system, encoding outer membrane protein, surface glycoconjugates and secretory system [19–22]. ompA exists in outer membrane vesicles and plays a role in cell adhesion and invasion [43]. basJ is involved in the synthesis of the *A. baumannii* iron carrier Acinetobacter. The ability of *A. baumannii* to compete for iron ions with the host is related to the pathogenicity and virulence of *A. baumannii* [44]. plc D is involved in the process of *A. baumannii* invading cells. Some studies have shown that plc D1, plc D2 and plc D3 cooperate to promote the bacterial invasion of host cells [45]. In this study, the detection rates of the ompA, basJ and plc D were 100%, which may be one of the reasons for the poor treatment of children infected with CRAB. The motility of bacteria is closely related to their ability to cause disease. Biofilms can resist the killing effect of antibiotics, resulting in bacterial drug resistance. csuA mainly plays a role in the early stage of biofilm formation, while abaI plays a role in the middle and late stages. pgaA promotes the polymerization of the main components of the extracellular matrix β-1-6-n-acetylglucosamine transmembrane outlet [46]. adeH is involved in coding the protein of the resistance-nodulation-cell division superfamily of active outflow systems, which is related to the drug resistance of *A. baumannii* to antibiotics [47]. In this study, the detection rates of 4 genes related to biofilm formation were more than 95%, the detection rates of csuA and abaI were 100%, and the those of adeH and pgaA were both 98.70%. Some studies have shown that the biofilm forming ability of drug-resistant isolates is weaker than that of relatively drug-sensitive isolates, indicating that the acquisition of drug resistance weakens their biofilm forming ability, which may also weaken their pathogenicity [48]. The tyrosine kinase encoded by ptk promotes the synthesis of capsular polysaccharide. Capsular polysaccharides produce adhesin, which is conducive to bacterial colonization and protects bacteria from the natural immune defence of the host [49]. Compared with the other seven virulence genes, the detection rate of this gene was the lowest, 94.80%. An interesting discovery of this study was that 73 isolates carry 8 virulence genes, 3 isolates carry 7 virulence genes and 1 isolate carried 5 virulence genes. The isolate carrying 5 virulence genes was the only MDR-AB isolate, suggesting that bacterial drug resistance may be related to the number of virulence genes carried. However, this is only a conjecture, and further exploration and verification are needed.

In summary, as an important nosocomial infectious pathogen, CRAB has gradually increased its pathogenicity and drug resistance, making treatment more difficult. Clinicians and laboratories should deeply understand the change in drug resistance mode and pay attention to monitoring the change trend of virulence, which will help clinicians use drugs reasonably, control infection in time and save lives. However, there are some limitations in this study. Although the drug resistance and virulence of CRAB in the past five years have been analysed, there is a lack of in-depth exploration of the correlation between virulence and drug resistance, and it is necessary to understand the changes in virulence and drug resistance during the epidemic period of COVID-19. Therefore, we will pay more attention to the above limitations in a follow-up study.

## Electronic supplementary material

Below is the link to the electronic supplementary material.

Supplementary Material 1: Table S1 Genome sequence table

## Data Availability

The datasets used and/or analysed during the current study available from the corresponding author on reasonable request.

## Abbreviations

ICU: Intensive care unit
PCR: Polymerase chain reaction
CHSU: Children’s Hospital of Soochow Universit
CLSI: Clinical and Laboratory Standards Institute
CRAB: Carbapenem-resistant *Acinetobacter baumannii*
MDR-AB: Multidrug-resistant *Acinetobacter baumannii*
XDR-AB: Extensivelydrug-resistance *Acinetobacter baumannii*
PDR-AB: Pandrug-resistant *Acinetobacter baumannii*

## Declarations

### Disclaimer

The views, opinions, assumptions, or any other information set out in this article are solely those of the authors and should not be attributed to the funders or any other person connected with the funders.
