# Supplementary material for: Insight into carbapenem resistance and virulence of Acinetobacter baumannii from a children’s medical centre in eastern China
Source: Ann Clin Microbiol Antimicrob. 2022 Nov 5;21:47. doi: 10.1186/s12941-022-00536-0 (PMC9637306; doi:10.1186/s12941-022-00536-0)
Supplement: Supplementary file 1 — Supplementary Material 1: Table S1 Genome sequence table [file 12941_2022_536_MOESM1_ESM.pdf]

Supplementary materials

Table S1 Genome sequence table

Gene		Sequence（5'-3'）	Size of product (bp)
**Virulence gene**	ompA	F：CTGCTCCATTAGCTGCTGCT R：AGGCTTCAAGTGACCACCAAG	560
	adeH	F：CAACTGAATGAACTTGAACAG R：GCTGCGTTGACACTACTTGC	291
	csuA	F：GGAACTATAGATTTTGGTGAAGC R：ACCCTTAGATATACGACTACC	348
	pgaA	F：GCTAAAGATCAGTTGTCGAAG R：TTCAGCAAAGCTTTCGGCATC	360
	abaI	F：GTGGCTCAAGACAGAGAATC R：ACGTTCTACTCCAAGAGGAG	297
	basJ	F：TCATCAGAATTCCAAGGTGTGC R：TTCTAACCATTCAGCTTCAGC	300
	ptk	F：ATGAACCAAAATACTAATACCG R：GTGTATTCAGTTTTATATTCAG	386
	plcD	F：GCGCTTATTGGTGGGCGCAAT R：CTGAACGGTGGCTTGTTGATAATG	246
**Carbapenem -resistant gene**	*bla*OXA-23	F：TGTCATAGTATTCGTCGTT R：TTCCCAAGCGGTAAA	453
	*bla*OXA-24	F：TTTGCCGATGACCTT R：TAGCTTGCTCCACCC	175
	*bla*OXA-51	F：ATGAACATTAAAGCACTCTTACTT R：CTATAAAATACCTAATTGTTCTAA	825
	*bla*OXA-2	F：CGCTGTTCGTGATGAGTTCC R：ATCGGCGTTGCCATAGTC	210
	*bla*OXA-10	F：TCCGAGTATGCCGTAAATG R：GCTCTTGTTTGGACCGCTAT	822
	*bla*VIM	F：GTGGATCGGGTTGTAGTCG R：CCACCTTCAGCATCGTCA	215
	*bla*IMP	F：GAAGGCGTTTATGTTCATAC R：GTAAGTTTCAAGAGTGATGC	587
	*bla*BIC	F：TATGCAGCTCCTTTAAGGGC R：TCATTGGCGGTGCCGTACAC	508
	*bla*GIM	F：TCGACACACCTTGGTCTGAA R：TCATTGGCGGTGCCGTACAC	432
	*bla*AIM	F：CTGAAGGTGTACGGAAACAC R：GTTCGGCCACCTCGAATTG	445
	*bla*SPM	F：TATGCAGCTCCTTTAAGGGC R：ACATTATCCGCTGGAACAGG	271
	*bla*KPC	F: CGTCTAGTTCTGCTGTCTTG R: CTTGTCATCCTTGTTAGGCG	798
	*bla*NDM	F: GGTTTGGCGATCTGGTTTTC R: CGGAATGGCTCATCACGATC	621

Table S2 Details of CRAB resistance gene carrying in 77 isolates

Serial number of CRAB	*bla*OXA-23	*bla*OXA-51	*bla*VIM	*bla*IMP	*bla*NDM
NO.1	+	+	+	+	
NO.2	+	+	+	+	
NO.3	+	+	+	+	
NO.4	+	+	+	+	
NO.5	+	+	+	+	
NO.6	+	+	+	+	
NO.7	+	+	+	+	
NO.8	+	+	+	+	
NO.9	+	+	+	+	
NO.10	+	+	+	+	
NO.11	+	+	+	+	
NO.12	+	+	+	+	
NO.13	+	+	+		
NO.14	+	+	+	+	
NO.15	+	+	+	+	
NO.16	+	+	+		
NO.17	+	+	+		
NO.18	+	+	+		+
NO.19	+	+	+		+
NO.20	+	+	+		+
NO.21	+	+	+	+	+
NO.22	+	+	+		+
NO.23	+	+	+		+
NO.24	+	+	+	+	+
NO.25	+	+	+	+	+
NO.26	+	+	+	+	+
NO.27	+	+	+	+	+
NO.28	+	+	+	+	
NO.29	+	+	+	+	
NO.30	+	+	+	+	
NO.31	+	+	+		
NO.32	+	+	+	+	
NO.33	+	+	+	+	
NO.34	+	+	+		
NO.35	+	+	+	+	
NO.36	+	+	+	+	
NO.37	+	+	+	+	
NO.38	+	+	+	+	
NO.39	+	+	+	+	
NO.40	+	+	+	+	
NO.41	+	+	+	+	

NO.42	+	+	+	+	
NO.43	+	+	+		
NO.44	+	+	+	+	
NO.45	+		+	+	+
NO.46	+	+	+	+	+
NO.47	+	+	+	+	+
NO.48	+	+	+		+
NO.49	+	+	+	+	+
NO.50	+	+	+		+
NO.51	+	+	+	+	
NO.52	+	+	+	+	+
NO.53	+	+	+	+	
NO.54	+	+	+		
NO.55	+	+	+	+	+
NO.56	+	+	+	+	+
NO.57	+	+	+	+	+
NO.58	+	+	+	+	+
NO.59	+	+	+	+	+
NO.60	+	+	+	+	+
NO.61	+	+	+	+	+
NO.62	+	+	+	+	
NO.63	+	+	+	+	
NO.64	+	+	+	+	
NO.65	+	+	+		
NO.66	+	+	+	+	
NO.67	+	+	+		
NO.68	+	+	+	+	
NO.69	+	+	+		
NO.70	+	+	+		
NO.71	+	+	+		
NO.72	+	+	+		
NO.73	+	+	+		
NO.74	+	+	+		
NO.75	+	+	+		
NO.76	+	+	+		
NO.77	+	+	+		

*Notes: "+" represents the gene detected in the isolate.

Table S3 Details of CRAB virulence gene carrying in 77 isolates

Serial number of CRAB	ompA	adeH	csuA	pgaA	abaI	basJ	ptk	plcD
NO.1	+	+	+	+	+	+	+	+
NO.2	+	+	+	+	+	+	+	+
NO.3	+	+	+	+	+	+	+	+
NO.4	+	+	+	+	+	+	+	+
NO.5	+	+	+	+	+	+	+	+
NO.6	+	+	+	+	+	+	+	+
NO.7	+	+	+	+	+	+	+	+
NO.8	+	+	+	+	+	+	+	+
NO.9	+	+	+	+	+	+	+	+
NO.10	+	+	+	+	+	+	+	+
NO.11	+	+	+	+	+	+	+	+
NO.12	+	+	+	+	+	+	+	+
NO.13	+	+	+	+	+	+	+	+
NO.14	+	+	+	+	+	+	+	+
NO.15	+	+	+	+	+	+	+	+
NO.16	+	+	+	+	+	+	+	+
NO.17	+	+	+	+	+	+	+	+
NO.18	+	+	+	+	+	+	+	+
NO.19	+	+	+	+	+	+	+	+
NO.20	+	+	+	+	+	+	+	+
NO.21	+	+	+	+	+	+		+
NO.22	+	+	+	+	+	+		+
NO.23	+	+	+	+	+	+	+	+
NO.24	+	+	+	+	+	+	+	+
NO.25	+	+	+	+	+	+	+	+
NO.26	+	+	+	+	+	+	+	+
NO.27	+	+	+	+	+	+	+	+
NO.28	+	+	+	+	+	+	+	+
NO.29	+	+	+	+	+	+	+	+
NO.30	+	+	+	+	+	+	+	+
NO.31	+	+	+	+	+	+	+	+
NO.32	+	+	+	+	+	+		+
NO.33	+	+	+	+	+	+	+	+
NO.34	+	+	+	+	+	+	+	+
NO.35	+	+	+	+	+	+	+	+
NO.36	+	+	+	+	+	+	+	+
NO.37	+	+	+	+	+	+	+	+
NO.38	+	+	+	+	+	+	+	+
NO.39	+	+	+	+	+	+	+	+
NO.40	+	+	+	+	+	+	+	+
NO.41	+	+	+	+	+	+	+	+

NO.42	+	+	+	+	+	+	+	+
NO.43	+	+	+	+	+	+	+	+
NO.44	+	+	+	+	+	+	+	+
NO.45	+	+	+	+	+	+	+	+
NO.46	+	+	+	+	+	+	+	+
NO.47	+	+	+	+	+	+	+	+
NO.48	+	+	+	+	+	+	+	+
NO.49	+	+	+	+	+	+	+	+
NO.50	+	+	+	+	+	+	+	+
NO.51	+	+	+	+	+	+	+	+
NO.52	+	+	+	+	+	+	+	+
NO.53	+	+	+	+	+	+	+	+
NO.54	+	+	+	+	+	+	+	+
NO.55	+		+		+	+		+
NO.56	+	+	+	+	+	+	+	+
NO.57	+	+	+	+	+	+	+	+
NO.58	+	+	+	+	+	+	+	+
NO.59	+	+	+	+	+	+	+	+
NO.60	+	+	+	+	+	+	+	+
NO.61	+	+	+	+	+	+	+	+
NO.62	+	+	+	+	+	+	+	+
NO.63	+	+	+	+	+	+	+	+
NO.64	+	+	+	+	+	+	+	+
NO.65	+	+	+	+	+	+	+	+
NO.66	+	+	+	+	+	+	+	+
NO.67	+	+	+	+	+	+	+	+
NO.68	+	+	+	+	+	+	+	+
NO.69	+	+	+	+	+	+	+	+
NO.70	+	+	+	+	+	+	+	+
NO.71	+	+	+	+	+	+	+	+
NO.72	+	+	+	+	+	+	+	+
NO.73	+	+	+	+	+	+	+	+
NO.74	+	+	+	+	+	+	+	+
NO.75	+	+	+	+	+	+	+	+
NO.76	+	+	+	+	+	+	+	+
NO.77	+	+	+	+	+	+	+	+

*Notes: “+” represents the gene detected in the isolate.
